# Short Interval Infield Sarcoma Development following Resection of Glioblastoma and Adjuvant Radiotherapy and Temozolomide

**DOI:** 10.1155/2013/591272

**Published:** 2013-08-29

**Authors:** Fahad E. Alotaibi, Kevin Petrecca

**Affiliations:** Department of Neurology and Neurosurgery, Montreal Neurological Institute and Hospital, McGill University and Department of Neurosurgery, McGill University Health Centre, 3801 University Avenue, 109 Montreal, QC, Canada H3A 2B4

## Abstract

*Background*. The development of 2 unassociated brain cancers in the same patient is a rare occurrence. Secondary cancers are generally thought to develop as an oncogenic consequence of the radiation therapy delivered to treat the primary cancers, always requiring a significant time interval between radiation treatment and secondary cancer development. *Case Description*. We report the development of an undifferentiated myxoid sarcoma only 13 months following radiation therapy for a glioblastoma. *Conclusion*. This case represents the shortest time interval reported between radiation therapy and secondary brain cancer development.

## 1. Introduction

A well-established long-term complication of cranial radiation is the development of secondary tumors. These tumors are typically meningiomas, but gliomas, ependymomas, medulloblastomas, sarcomas, schwannomas, and primitive neuroectodermal tumors have been reported [1]. Most commonly, a long interval is required between the time of radiation delivery and the appearance of the second tumor, often decades in the case of meningiomas. We report the development of an undifferentiated myxoid sarcoma only 13 months following radiation therapy and temozolomide for a glioblastoma.

## 2. Case Description

**Figure 1:**
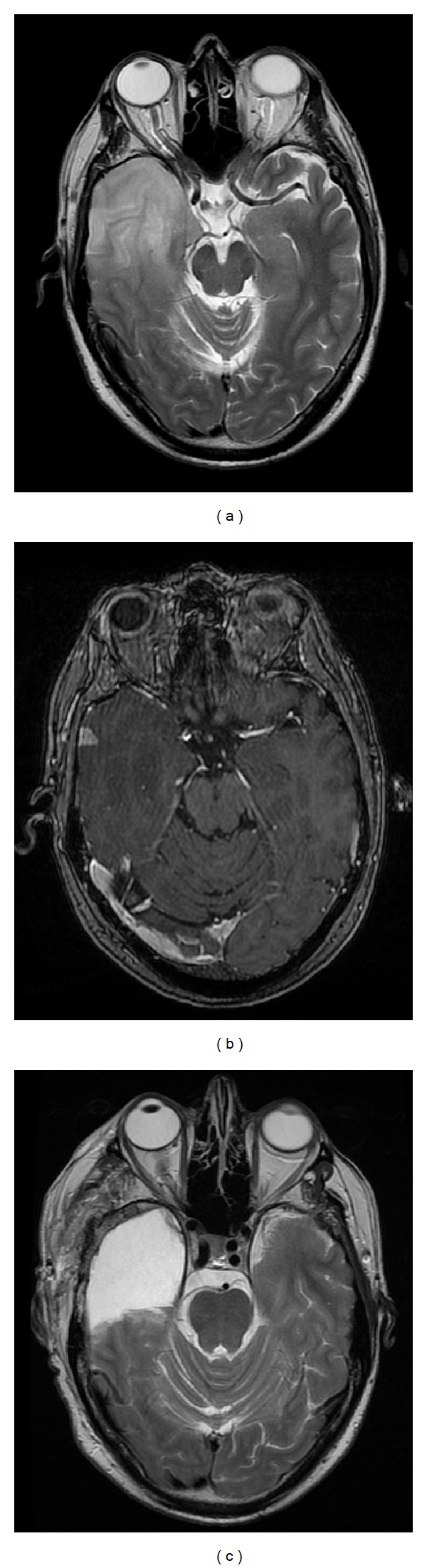
Pre- and Post-Operative Glioblastoma Imaging. (a) Pre-operative axial T_2_-weighted image. (b) Pre-operative gadolinium-enhanced T_1_-weighted image. (c) Post-operative axial T_2_-weighted image.

A 47-year-old man presented with a seizure. Magnetic resonance imaging (MRI) revealed a lesion involving the right temporal lobe that was hyperintense on T_2_-weighted sequences with a small area of gadolinium enhancement, consistent with a malignant glioma (Figures 1(a) and 1(b)). The patient had no personal cancer history and no family history of a cancer syndrome. He underwent resection (Figure 1(c)) and the lesion was classified as a World Health Organization glioblastoma (GBM). The cancer exhibited hypermethylation at the O^6^-methylguanine-DNA methyltransferase promoter region, was wild-type at the IDH 132 position, and the epidermal growth factor receptor was amplified. Adjuvant therapy consisted of radiotherapy (60 Gy) and chemotherapy according to the Stupp protocol [2]. Maintenance temozolomide treatment continued for 12 months.

**Figure 2:**
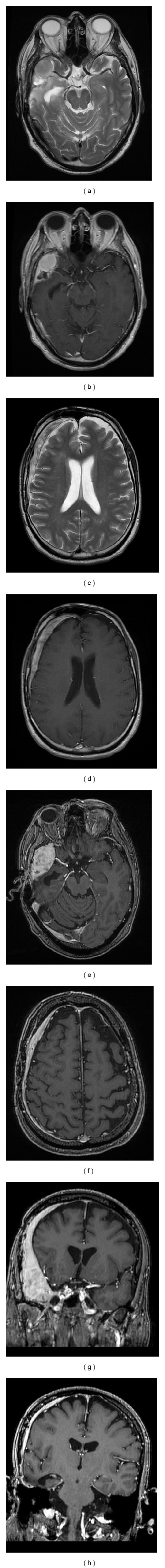
Appearance and progression of secondary tumor. T_2_-weighted ((a) and (c)) and gadolinium enhanced T_1_-weighted ((b) and (d)) images demonstrating the first appearance of the secondary tumor. ((e)–(h)) Gadolinium enhanced T_1_-weighted images showing progression of secondary tumor.

One month later, a surveillance MRI revealed a new lesion within the resection cavity and extending over the right frontal convexity that appeared to be contained within the subdural extraleptomeningeal space (Figures 2(a)–2(d)). The lesion, which was iso- to hypointense on T_2_-weighted sequences and homogenously enhancing on gadolinium-enhanced T_1_-weighted sequences, was consistent with a hematoma or an atypical recurrent GBM. There was no evidence of recurrent GBM within the brain. Repeat imaging 2 months later demonstrated progression of the lesion over the entire right hemisphere (Figures 2(e)–2(h)).

**Figure 3:**
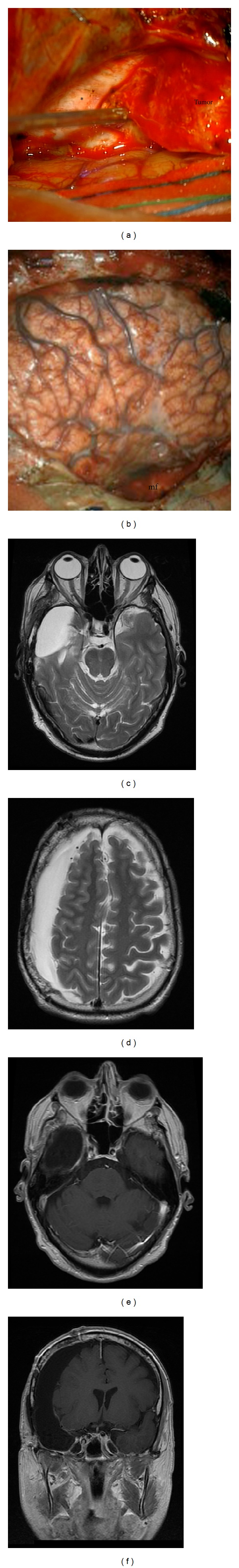
Intraoperative findings and postoperative results. (a) Intraoperative image showing subdural extra-leptomeningeal tumor. Asterisk indicates anterior fossa dura; mf: middle fossa. (b) Intraoperative image revealing complete resection. T_2_-weighted ((c) and (d)) and gadolinium enhanced T_1_-weighted ((e) and (f)) images showing no evidence of sarcoma recurrence at 3 months.

A second craniotomy was performed which revealed a solid, firm tumor within the subdural and extra-leptomeningeal space. A clear dissection plane existed between the tumor and the pia-arachnoid (Figure 3(a)), and a complete resection was achieved (Figure 3(b)). This tumor was classified as an undifferentiated myxoid sarcoma based on the lack of a glial proliferation, strong CD68 and vimentin immunolabeling in the majority of tumor cells and the absence of CD1a and GFAP immunolabeling. The patient went on to receive radiotherapy to the resection margins followed by doxorubicin chemotherapy. An MRI performed 3 months following the second surgery revealed no evidence of recurrent sarcoma (Figures 3(c)–3(f)).

## 3. Discussion

Tumors that have been reported to develop following cranial radiation include meningiomas, gliomas, sarcomas, and medulloblastomas [1–17]. The time interval between radiotherapy and the occurrence of these tumors ranges from 4 to 47 years, with a mean interval of 18.8 years. Chowdhary et al. [1] reported a significant positive correlation between the time interval following radiotherapy and the development of a meningioma (21.8 years) or a sarcoma (7.7 years). 

The coincidence of a GBM and an intracranial sarcoma in the same patient is rare; only 2 cases have been reported. A 9-month-old with a GBM treated with surgery and chemotherapy, but not radiotherapy, developed an intracranial fibrosarcoma 5 years later [7]. The second, a 43-year-old with an intraparenchymal fibrosarcoma treated with surgery followed by fractionated conformal radiotherapy developed a recurrence 3 months later and was treated with stereotactic radiosurgery. Nine months later the lesion progressed and a second surgery was performed. The lesion was classified as a GBM [18]. 

One case of intracranial myxoid sarcoma has been reported, a metastasis from primary lung lesion [19]. There are no reports suggesting an association between temozolomide therapy and the development of myxoid sarcomas. 

The case described here is the shortest time interval reported between the completion of radiotherapy (13 months) and the development of a second malignancy. This short latency suggests that prior radiotherapy may not be the cause of this undifferentiated myxoid sarcoma. An alternate hypothesis is that this patient carries an underlying genetic disorder predisposing to cancer development.
